# Gas Phase Oxidation of Carbon Monoxide by Sulfur Dioxide Radical Cation: Reaction Dynamics and Kinetic Trend With the Temperature

**DOI:** 10.3389/fchem.2019.00140

**Published:** 2019-03-26

**Authors:** Daniele Catone, Mauro Satta, Antonella Cartoni, Mattea C. Castrovilli, Paola Bolognesi, Stefano Turchini, Lorenzo Avaldi

**Affiliations:** ^1^Istituto di Struttura della Materia, Consiglio Nazionale Delle Ricerche (CNR-ISM), Area della Ricerca di Roma Tor Vergata, Rome, Italy; ^2^Istituto per lo Studio dei Materiali Nanostrutturati (CNR-ISMN), Dipartimento di Chimica, Sapienza Università di Roma, Rome, Italy; ^3^Dipartimento di Chimica, Sapienza Università di Roma, Rome, Italy; ^4^Istituto di Struttura della Materia, Consiglio Nazionale Delle Ricerche (CNR-ISM), Area della Ricerca di Roma 1, Rome, Italy

**Keywords:** rate constants, temperature, VTST, synchrotron radiation, oxidation, reaction dynamics

## Abstract

Gas phase ion chemistry has fundamental and applicative purposes since it allows the study of the chemical processes in a solvent free environment and represents models for reactions occurring in the space at low and high temperatures. In this work the ion-molecule reaction of sulfur dioxide ion ${\text{SO}}_{2}^{.+}$ with carbon monoxide CO is investigated in a joint experimental and theoretical study. The reaction is a fast and exothermic chemical oxidation of CO into more stable CO_2_ by a metal free species, as ${\text{SO}}_{2}^{.+}$, excited into ro-vibrational levels of the electronic ground state by synchrotron radiation. The results show that the reaction is hampered by the enhancement of internal energy of sulfur dioxide ion and the only ionic product is SO^.+^. The theoretical approach of variational transition state theory (VTST) based on density functional electronic structure calculations, shows an interesting and peculiar reaction dynamics of the interacting system along the reaction path. Two energy minima corresponding to [SO_2_–CO]^.+^ and [OS–OCO]^.+^ complexes are identified. These minima are separated by an intersystem crossing barrier which couples the bent ^3^B_2_ state of CO_2_ with C_2v_ symmetry and the ^1^A_1_ state with linear D_∞h_ symmetry. The spin and charge reorganization along the minimum energy path (MEP) are analyzed and eventually the charge and spin remain allocated to the SO^.+^ moiety and the stable CO_2_ molecule is easily produced. There is no bottleneck that slows down the reaction and the values of the rate coefficient *k* at different temperatures are calculated with capture theory. A value of 2.95 × 10^−10^ cm^3^s^−1^molecule^−1^ is obtained at 300 K in agreement with the literature experimental measurement of 3.00 × 10^−10^ ± 20% cm^3^s^−1^molecule^−1^, and a negative trend with temperature is predicted consistently with the experimental observations.

## Introduction

The oxidation of carbon monoxide into carbon dioxide is a challenging topic in chemistry as well as the oxidation of other simple molecules as methane and alcohols (Ten Brink et al., 2000; Guo et al., 2014; Schwarz et al., 2017). CO has a little dipole moment μ_D_ of 0.112 D with a partial negative charge on C, the bond length and the polarizability are, respectively 1.13 Å and 1.953 Å^3^ (Linstrom and Mallard, 2005). Carbon monoxide is one of the most common environmental pollutants, mainly produced by human activities. It is not a green-friendly molecule due to its high toxicity and many efforts have been devoted to efficiently transform it into CO_2_ with molecular oxygen O_2_. The oxidation is thermodynamically favored but kinetically demanding and relative high temperature and metal catalysts are used. Different metal catalysts have been studied both in solution and at the interface of a solid phase: a growing activity is directed toward the achievement of reactions at room temperature, which represents a more economic solution (Wu et al., 2014; Zhu et al., 2015). Recently, the role of cations in the catalytic converters has been demonstrated to be fundamental for the oxidation at low temperatures (Peterson et al., 2014). At the microscopic level the oxidation corresponds to an “O” atom transfer to CO molecule and in principle neutral or ionic species with high tendency to promote O-transfer should be able to oxidize CO (Δ${\text{H}}_{\text{f}}^{∙}=$ −26.42 kcal mol^−1^) into more stable CO_2_ (Δ${\text{H}}_{\text{f}}^{∙}=$ −94.05 kcal mol^−1^) (Linstrom and Mallard, 2005). Although O_2_ is the most important oxidant due to its abundance in the Earth's atmosphere, other species, like ions or radical cations, can oxidize CO, also at room temperature and without catalysts (Anicich, 1993a,b; Bacskay and Mackie, 2005). The investigation of the dynamics of these processes is of fundamental interest and may be helpful to develop models for a more efficient, green-friendly and metal-free oxidation of CO (Crabtree, 2009). Moreover, these reactions could also play a role in the chemistry of Interstellar medium and extra-terrestrial atmospheres as well as in Earth's atmosphere, mainly troposphere, where H_2_O, CO, CO_2_, NO_x_, O_3_, SO_2_ (present in fraction of ppm, depending on environmental conditions and geographical position, Speidel et al., 2007), and other reactive molecules are in their neutral and ionized forms due to corona discharge phenomena (Cacace and de Petris, 2000; Petrie and Bohme, 2007; Larsson et al., 2012). Recently, we have studied the dynamics of the reaction of hydrogen atom transfer (HAT) of ${\text{SO}}_{2}^{.+}$ radical cation with methane and water: the ${\text{HSO}}_{2}^{+}$ ions are formed with high reaction efficiencies in both reactions, but with different kinetic trends vs. temperature (Cartoni et al., 2017). In this work we focus our attention on sulfur dioxide, as the oxidant agent, i.e., source of oxygen atoms. Indeed, ${\text{SO}}_{2}^{.+}$ can be also a source of “O” in the reactions with neutrals which are more stable in their oxidized form, as in the CO case, since the Δ${\text{H}}_{\text{f}}^{∙}$ of ${\text{SO}}_{2}^{.+}$ and SO^.+^ is similar, being 213.0 and 239.2 kcal mol^−1^, respectively (Lias et al., 1988). This work reports the investigation of the oxidation of CO molecule in the gas-phase by the metal free radical cation sulfur dioxide:

(1) $$\begin{array}{l} {\text{SO}}_{2}^{.+}+\text{CO}\to {\text{SO}}^{.+}+{\text{CO}}_{2} \end{array}$$

The rate constant of this reaction, 3.00 × 10^−10^ ± 20% cm^3^s^−1^molecule^−1^, is known from literature at 300 K (Fehsenfeld and Ferguson, 1973), but to the best of our knowledge no experimental details or other data are reported. From the heats of formation (Δ${\text{H}}_{\text{f}}^{∙}$) the reaction results exothermic by 41.4 kcal mol^−1^ (Lias et al., 1988). Here the reaction dynamics has been explored theoretically by a combination of Density Functional Theory (DFT), Variational Transition State Theory (VTST) (Truhlar and Garrett, 1984; Bao and Truhlar, 2017) and capture theory, which allows to explore the minimum energy path (MEP) of the reaction and to obtain the rate constant as a function of temperature. The effects of spin and charge of the [SO_2_–CO]^.+^ complex along the reaction path have been evaluated and discussed to obtain mechanistic details of the process. Estimation of the reaction efficiency (ϕ = *k/k*_coll_), where *k* is the experimental rate constant and *k*_*coll*_ the collision rate (Bowers, 1979), has been obtained. The experimental study has been performed at the Elettra synchrotron (Trieste) using tunable VUV radiation to generate “hot” ${\text{SO}}_{2}^{.+}$ ions in vibrationally excited ionic ground state. The trend of the reaction as a function of photon energy has been measured and compared with the theoretical calculations. This study provides important mechanistic details of the reaction that, in perspective, can be proposed as a possible alternative to be explored for the oxidation of carbon monoxide and for the removal of CO, produced both by human and natural activities, from the lower atmosphere. This is a hot topic in environmental science, since the reaction with oxygen is quite slow and the oxidation with the hydroxyl radical OH, despite seeming to be the main route for the tropospheric CO elimination, is not the only operative mechanism (Jaffe, 1968; Badr and Probert, 1995; Carpenter and Nightingale, 2015). All along the paper the radical symbol ‘^.^’ has been omitted for the sake of simplicity.

## Materials and Methods

### Materials

All the samples were used at room temperature. Sulfur dioxide was purchased from Sigma-Aldrich with a purity >99.98%, whereas CO is from SIAD with purity >99.99%. The security of CO gas line has been continuously checked with the Carbon Monoxide Alarm Ei204EN model from Ei Electronics (Ireland).

### Experimental Section

The ion-molecule reaction of ${\text{SO}}_{2}^{+}$ with CO has been investigated at the “Circular Polarization” beamline (CiPo) of ELETTRA synchrotron (Trieste, Italy). The characteristics and the experimental performance of the CiPo beamline at ELETTRA as well of the experimental set-up are reported in previous works (Derossi et al., 1995; Cartoni et al., 2014, 2017, 2018; Castrovilli et al., 2014; Satta et al., 2015a). Briefly, the beamline is provided with an electromagnetic elliptical undulator/wiggler and a Normal Incidence Monochromator to cover the 8–40 eV energy range. The aluminum grating, with a resolving power of about 1,000, was used for the experiment in the energy range 8–17 eV. The ${\text{SO}}_{2}^{+}$ ions are produced from SO_2_ in the ion source (pressure of about 5.0 × 10^−6^ mbar) and transported into an octupole, where they react with the neutral reagent CO at a pressure in the range of 10^−6^–10^−5^ mbar. The experiment has been done by recording the ion yields of ionic reagent and products as a function of the photon energy, hν, between 12 and 15 eV (energy step = 100 meV and dwell time = 5 s/point) at the fixed pressure of the neutral reagent (about 10^−6^–10^−5^ mbar) and nominal collision energy (CE) zero. The CE = 0 eV is determined by measuring the ${\text{SO}}_{2}^{+}$ yield as a function of the retarding field at the entrance of the octupole. An average energy spread of 100 meV was evaluated. The mass spectrum resulting from this ion-molecule reaction has been acquired in the mass over charge range 10<m/z<70 (dwell time of 2s/point) at the photon energy hν = 14.0 eV and CE = 0 eV. No impurities were detected. The production of a very little amount of SO^+^ in the ion source, due to the second order radiation, has been observed and considered in data analysis. SO^+^ ions do not react with CO as also reported in the literature (Anicich, 1993a,b). The reaction efficiencies were evaluated by calculating the experimental ratio of product/reagent ion intensity (SO^+^/${\text{SO}}_{2}^{+}$), and using statistical propagation error formula to estimate the error bars. Data analysis has been performed using OriginPro8 program.

### Theoretical Section

The optimal choice of the computational level for the energetic and dynamic description of the title reaction has been a subtle task due to the difficulty to concurrently reproduce the correct magnitude and direction of the electric dipole moment of CO, and the enthalpies of the oxygen-breaking reactions CO_2_ → CO + O(^3^P) and ${\text{SO}}_{2}^{+}\to {\text{SO}}^{+}$ + O(^3^P). The energetic of these dissociation reactions has to be correctly described in order to obtain a reasonable MEP along which the oxygen transfer takes place. Several levels of ab-initio methods, including Second-Order Møller-Plesset Perturbation Theory MP2 (Head-Gordon et al., 1988), Becke 3-Parameter (Exchange), Lee, Yang and Parr B3LYP (Lee et al., 1988) and Minnesota Functionals M06L (Zhao and Truhlar, 2006) with different basis sets, have been tested. The τ (kinetic energy density) dependent functional Voorhis and Scuseria's kinetic-energy-dependent exchange–correlation, VSXC (van Voorhis and Scuseria, 1998), produces reasonable energetic data when applied to open shell molecules (Johansson, 2006; Gao et al., 2013; Gao and Li, 2014). The VSXC has demonstrated the ability to reproduce both the CO electric dipole moment and reaction enthalpies with the basis set Augmented Triple-zeta correlation-consistent polarized, aug-cc-pvtz (Dunning, 1989) on sulfur and carbon atoms, and cc-pvtz on the three oxygen atoms. The electric dipole moment of CO has been calculated as 0.189 D which is in agreement with the experimental value of 0.112 D (Linstrom and Mallard, 2005), with the electric negative pole correctly located on the carbon atom. The enthalpies of the oxygen dissociation from CO_2_ and ${\text{SO}}_{2}^{+}$ have been calculated, 131.9 and 86.2 kcal mol^−1^ respectively, while the corresponding experimental values are 127.3 and 84.4 kcal mol^−1^ (Linstrom and Mallard, 2005), with computational errors below 5%. The calculation of the reaction enthalpy of the title reaction gives 45.7 kcal mol^−1^, which is in agreement with the experimental value of 41.4 kcal mol^−1^ (Lias et al., 1988). All these calculations have been corrected by the zero point energies (ZPE), with the underlying harmonic vibrational frequencies scaled by the coefficient 0.986 (Alecu et al., 2010).

The MEP of the reaction has been studied by partial geometrical optimization of all the nuclear degrees of freedom except the molecular coordinates over which the scan has been performed. The symmetry of the system and the barrierless nature of this reaction do not allow for the use of a single coordinate to describe the whole MEP over which the exothermic reactive process occurs. The reaction has been divided in three sections: the first one considers the initial evolution with the reactants coming together and forming the initial complex (the scanning coordinate is S-C distance); the second section involves the transformation of the initial complex into a second, more stable one (here the scan has been performed over C-O distance); the third part of the MEP follows the reaction from this more stable complex to the products region (the scanning coordinate is the S-O distance). For a better understanding of the construction of the MEP see also the section Results and Discussion. The charge and spin population are based on the Mulliken analysis of the electron density (Mulliken, 1955). All the quantum chemical calculations were performed with the Gaussian09 package (Frisch et al., 2016).

The MEP has been used to compute the total molecular partition functions [Q(T)] of the reactive complex [SO_2_–CO]^+^ in the range of temperatures 300–6,000 K. Within the Variation Transition State Theory (VTST) (Bao and Truhlar, 2017) these partition functions are used to localize the kinetic bottleneck of the reactive flux of trajectories moving along the MEP. The relative structure will be discussed in the following sections together with the temperature dependent Langevin rate coefficient modified and parametrized for the ion-polar molecules reactions (Su, 1994). This rate coefficient depends on the polarizability and dipole moment of the neutral reagent molecule, which we have calculated at the same level of theory described above, and whose values in atomic units are, respectively 12.86 and 0.0744. The electron charge and spin densities have been described by surfaces with isovalue of 0.2 au and 0.02 au, respectively.

## Results and Discussion

**Figure 1:**
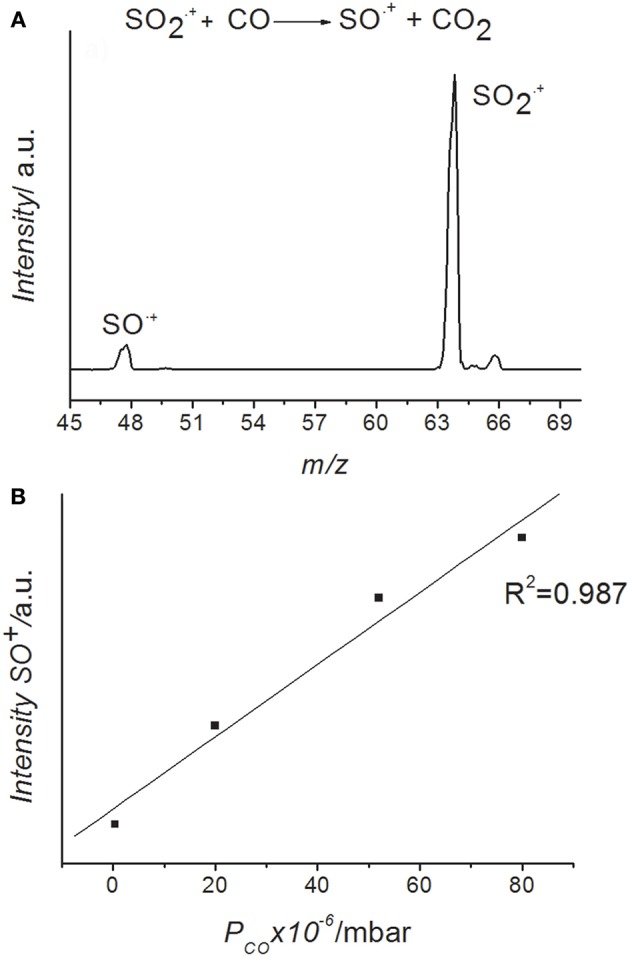
**(A)** Mass spectrum recorded at the photon energy of 14.0 eV, nominal CE = 0 eV and P_CO_ = 5.0 × 10^−5^mbar. **(B)** Trend of SO^+^ ion yield vs. CO pressure in the ${\text{SO}}_{2}^{+}$/CO ion-molecule reaction. The SO_2_ pressure inside the ion source was 4.6 × 10^−6^ mbar.

The photoelectron spectrum of SO_2_ reported in the literatures (Wang et al., 1987; Holland et al., 1994; Li et al., 2004) shows two bands in the energy range 12-15 eV, the ground X ^2^A_1_ state (12.349 eV) and two excited electronic states: ^2^B_2_ (12.988 eV) and ^2^A_2_ (13.338 eV) very close in energy. As already widely discussed in our previous work (Cartoni et al., 2017) the ${\text{SO}}_{2}^{+}$ ions produced in these excited states decay to excited ro-vibrational levels of the electronic ground state of the ion in a time scale of about 10 ns (Dujardin and Leach, 1981; Lévêque et al., 2014), i.e., shorter than the few ms needed to reach the reaction zone, where the interaction with the CO molecule occurs. Moreover, as checked in our measurements and demonstrated by a photoelectron-photoion coincidence (PEPICO) study (Brehm et al., 1973) the ${\text{SO}}_{2}^{+}$ ions do not dissociate in the energy range explored in this work. Following these experimental evidences the reagent ${\text{SO}}_{2}^{+}$ is considered a hot ion in its electronic ground state with an increasing internal energy as photon energy increases from the ionization threshold of SO_2_ at 12.349 eV (Linstrom and Mallard, 2005). The mass spectrum obtained by the ion-molecule reaction between ${\text{SO}}_{2}^{+}$ and CO is shown in Figure 1A, while the mass spectrum of SO_2_ alone, before the reaction, is shown in the supplementary material (Figure 1S).

**Figure 2:**
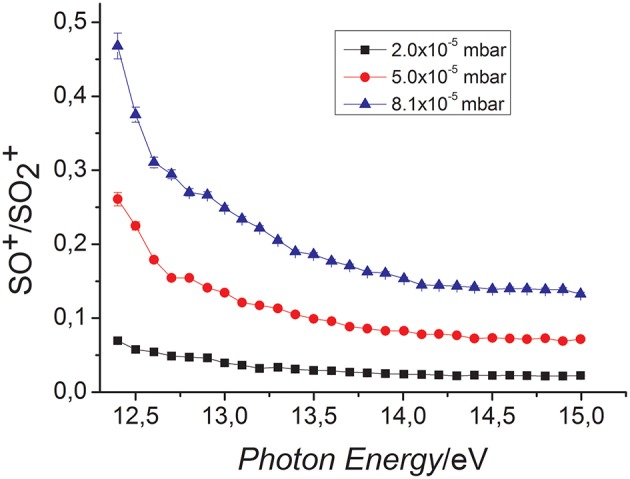
SO^+^/${\text{SO}}_{2}^{+}$ ratio vs. photon energy in the reaction of ${\text{SO}}_{2}^{+}$ with CO at the nominal CE = 0 eV and different CO pressures: 2.0 × 10^−5^ mbar (black line), 5.0 × 10^−5^ mbar (red line), 8.1 × 10^−5^ mbar (blue line).

The only ionic product observed is the ion SO^+^ at m/z 48 produced by the “oxygen-transfer” from ${\text{SO}}_{2}^{+}$ to CO. Accordingly, the CO_2_ molecule is the neutral counterpart generated in this reaction. In Figure 1B the SO^+^ ion intensity as function of CO pressure at the photon energy 14.0 eV is reported. The trend shows that SO^+^ is produced by reaction (1). The yields of the reagent ${\text{SO}}_{2}^{+}$ and product SO^+^ ions have also been recorded as function of photon energy and different fixed pressures of reagent gas, CO. The SO^+^/${\text{SO}}_{2}^{+}$ ratios vs. photon energy are reported in Figure 2.

The results show clearly that, at all pressures, the reaction is not favored by the increased internal energy of ${\text{SO}}_{2}^{+}$, suggesting the formation of a possible weakly bound complex between ${\text{SO}}_{2}^{+}$ and CO and a very fast process. To the purpose the reaction efficiency, ϕ, relative to the collision rate (*k*_*coll*_) (Su and Chesnavich, 1982; Su, 1994) of the reaction was estimated considering the experimental kinetic constant *k* = 3.00 × 10^−10^ ± 20% cm^3^s^−1^molecule^−1^ at 300 K (Fehsenfeld and Ferguson, 1973). The calculated ϕ values were around 1 considering both the *k*_*coll*_ = 2.81 × 10^−10^ cm^3^s^−1^molecule^−1^ (Su and Chesnavich, 1982) and 2.95 × 10^−10^ cm^3^s^−1^molecule^−1^ (Su, 1994) at 300 K, the latter model being more accurate. The high reaction efficiency demonstrates the effectiveness of this reaction.

To explore the reaction dynamic of this interesting and apparently simple system, a challenging and complete theoretical study has been performed.

**Figure 3:**
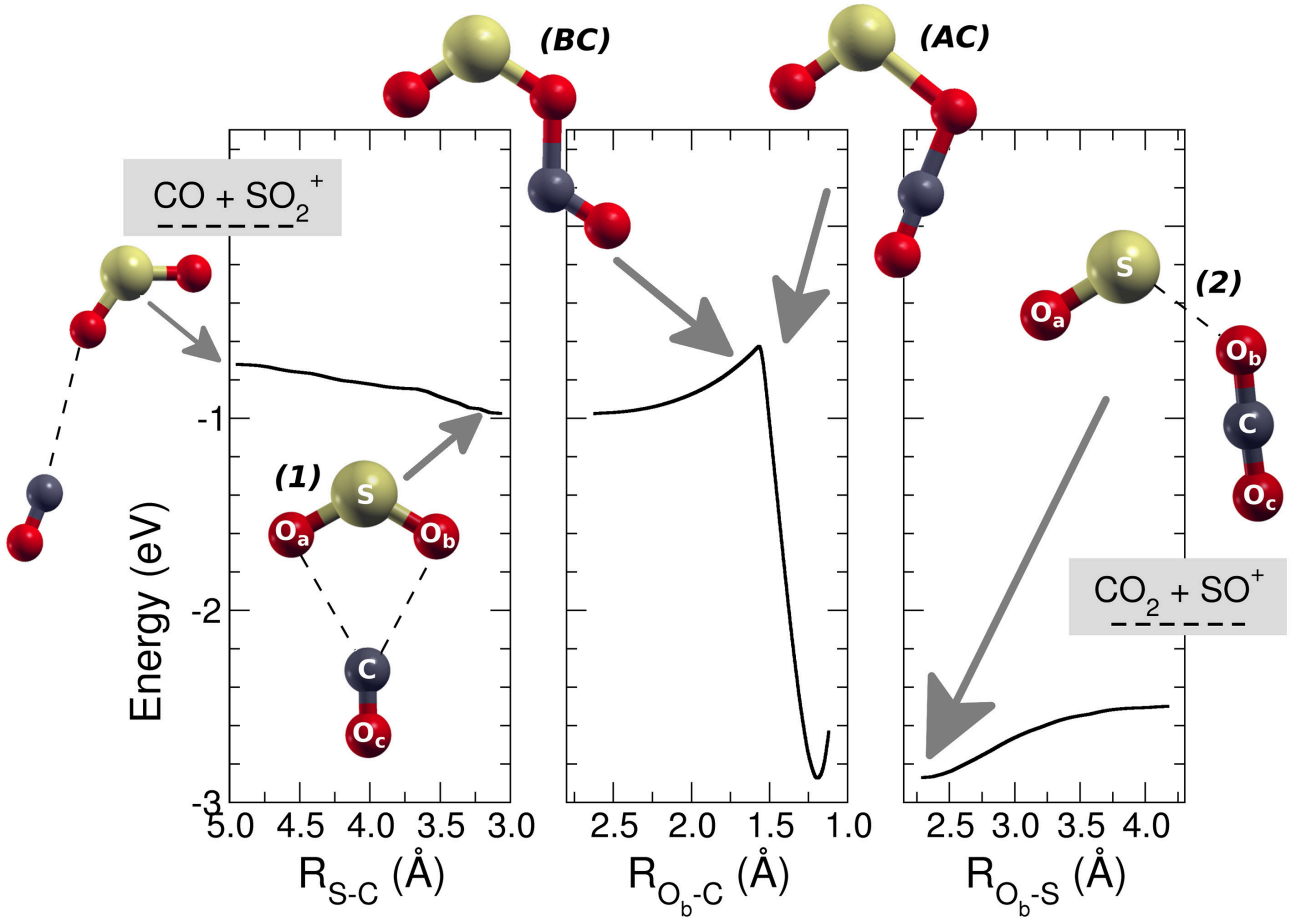
Minimum energy path for the reaction ${\text{SO}}_{2}^{+}$ + CO → SO^+^ + CO_2_. On the left part of the figure the approaching region from the reactants (leftmost structure) is reported together with the structure of the first complex [SO_2_–CO]^+^ *(1)*. In the central panel the barrier, corresponding to the intersystem crossing is presented with the structure of the molecular system just before (BC) and after the crossing (AC). In the right part of the figure the region of the products and the picture of the structure of the most stable adduct *(2)* [OS–OCO]^+^ is reported.

The MEP is characterized by a barrier connecting two minima corresponding to a first reagent complex [SO_2_–CO]^+^ ***1***, and a second more stable product complex [OS–OCO]^+^ ***2*** (Figure 3). This energy barrier is well-below the energy of the reagents, and hence the entire reaction results to be barrierless. The labels of the oxygen atoms used trough this discussion are those presented in Figure 3 where the O_a_ is the inactive spectator oxygen bound to the S atom, O_b_ is the atom transferred from the S to the C atom, and the O_c_ is the inactive atom bound to the carbon atom.

The MEP connecting the asymptotic energy levels of the ${\text{SO}}_{2}^{+}$ and CO reagents with those of the products has been divided in three parts (see Figure 3): (*i*) from the reagents to the first complex ***1*** [SO_2_–CO]^+^, where the scanning coordinate is the S-C distance; (*ii*) the second part of the MEP connects the first complex ***1*** to the second one [OS–OCO]^+^ ***2***, and the scanning coordinate is the O_b_-C distance; (*iii*) the last section of the MEP describes the system from the second complex ***2*** to the final products SO^+^ and CO_2_, with the O_b_-S distance as scanning coordinate. The first complex, [SO_2_–CO]^+^ ***1***, has a C_2v_ symmetry and an energy of −0.974 eV (−22.5 kcal mol^−1^) with respect to the energy of the reagents (which is the energy reference for all the energies given therein). The S-C distance is 3.1 Å, the O_b_ and C atoms are 2.7 Å apart and the OSO angle of 123.8 deg (see also Table 1).

**Table 1:** Main geometrical parameters of the adduct ions of the [SO_2_/CO]^.+^ system showed in Figure 3 along the minimum energy path, calculated at the DFT level of theory (see main text for further details).

	**Complex *1***	**BC**	**AC**	**Complex *2***
R(SO_a_)	1.5	1.4	1.5	1.4
R(SO_b_)	1.5	1.6	2.0	2.3
R(CO_b_)	2.7	1.6	1.5	1.2
R(CO_c_)	1.1	1.1	1.1	1.2
θ(O_a_SO_b_)	123.8	114.2	106.2	112.3
θ(SO_b_C)	89.9	121.3	106.7	131.4
θ(O_b_CO_c_)	151.6	121.5	178.9	178.2

*R is in Å and θ in deg*.

**Figure 4:**
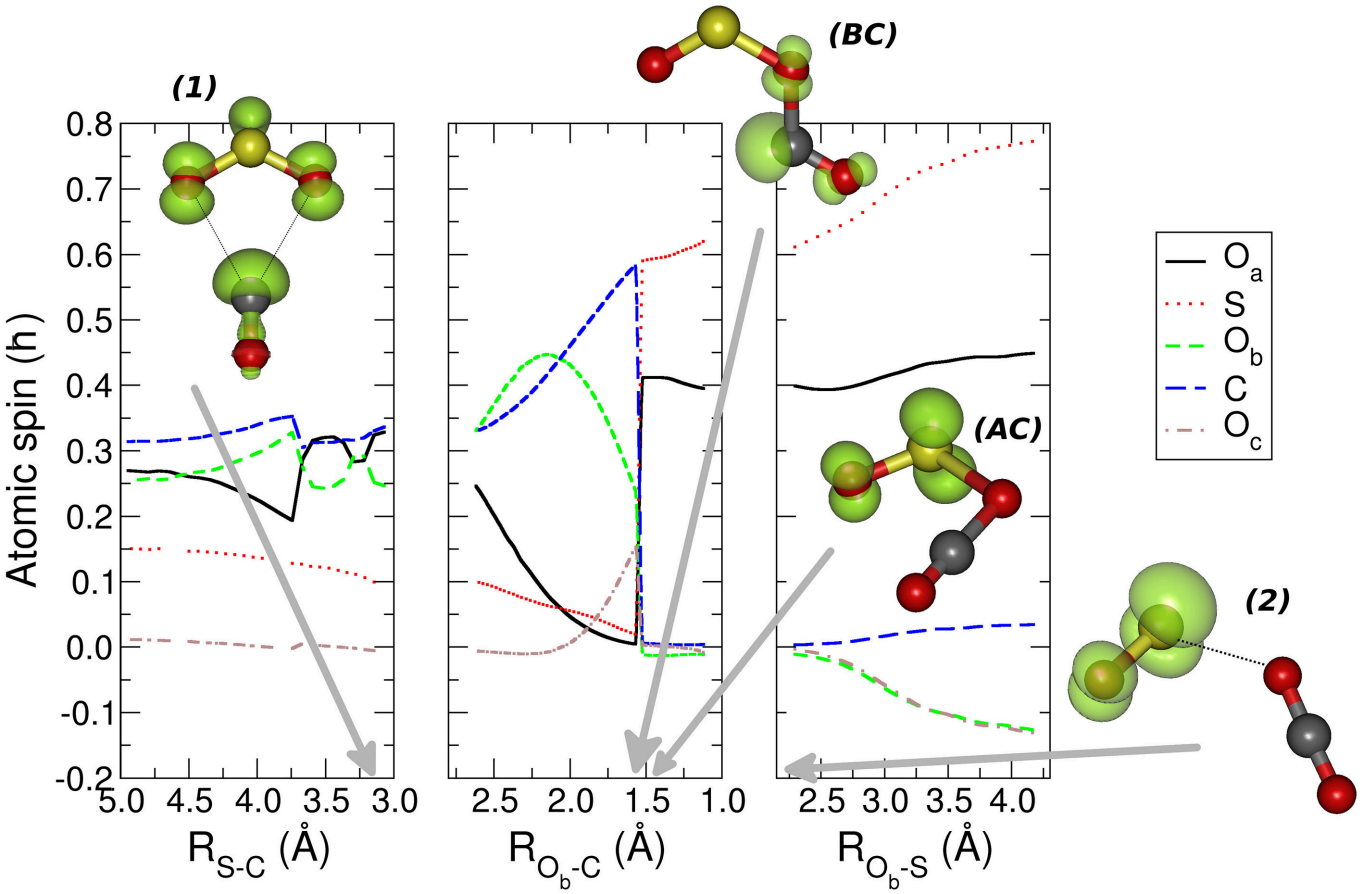
The atomic spin Mulliken population is presented for the nuclei of the molecular adducts along the coordinates that describe the MEP. The structures represent the most relevant molecular complexes during the reaction: the first minimum complex *(1)*, the structures before (BC) and after (AC) intersystem crossing, and second complex *(2)* are presented. The green isosurfaces represent the spin density surface around the atoms participating in the reaction. The gray arrows indicate the values of the different coordinates corresponding to the four relevant molecular structures. For further details see the main text.

**Figure 5:**
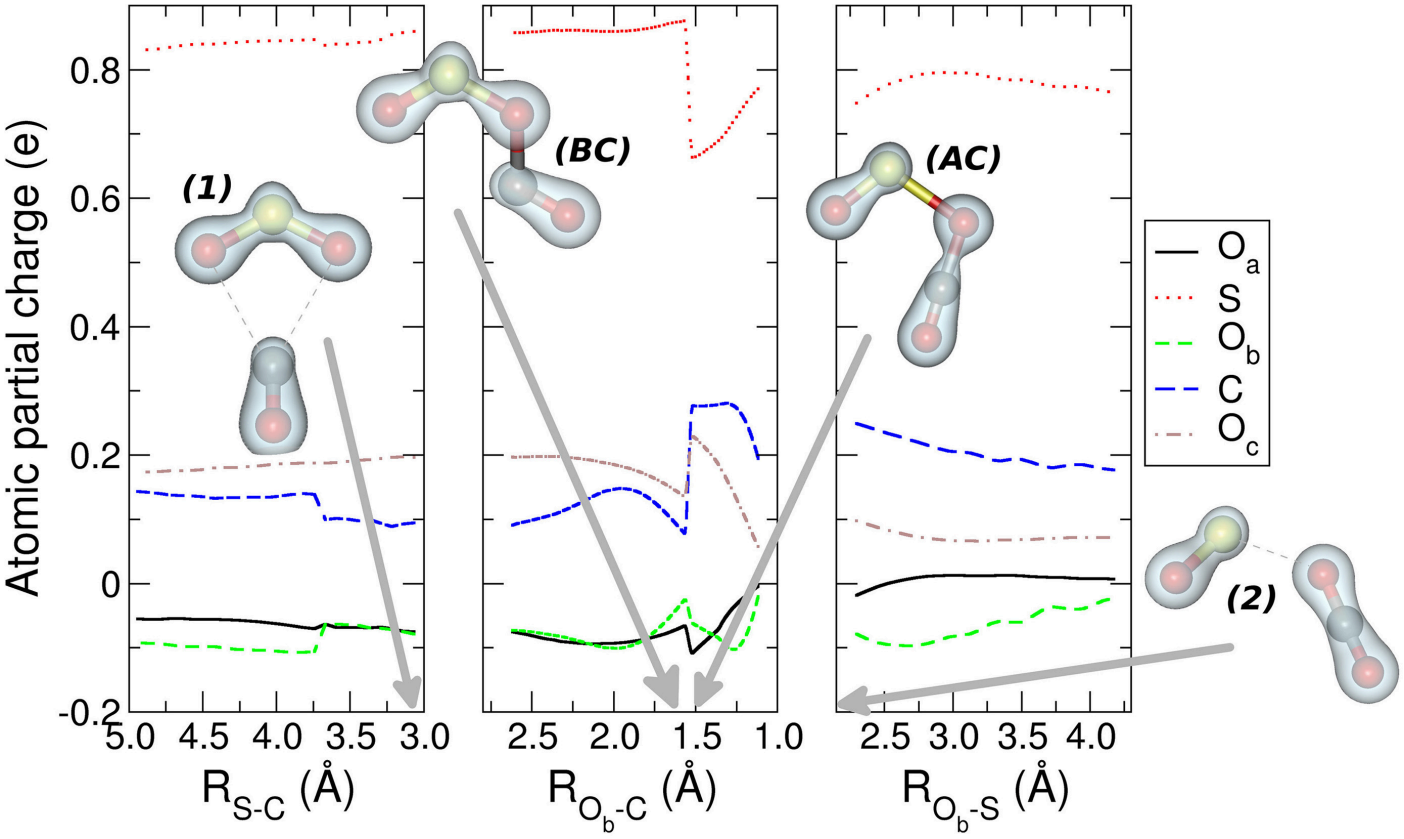
The Mulliken atomic partial charges of the five nuclei participating to the reaction is reported for the three regions of the MEP leading the reactants to the products. The structures and the electronic isodensity surfaces of the first minimum complex (1), structures before (BC) and after (AC) intersystem crossing, and second complex (2) are presented. The gray arrows indicate the values of the different coordinates corresponding to the four relevant molecular structures. For further details see the main text.

In the central panel of Figure 3 it is represented the region of the MEP with the barrier, at an energy of −0.626 eV (−14.4 kcal mol^−1^), which leads the system to the region of the products, by passing through an intersystem crossing point which connects the ^1^A_1_ and ^3^B_2_ lower electronic energy levels of CO_2_ (Zhou et al., 2013 see Figures 2S, 3S in the supplementary material for the discussion of the angle dependence OCO over the triplet and singlet ground states for CO_2_ alone and OCO inside the reacting system). The minimum energy geometry of the ^3^B_2_ state of CO_2_ has a C_2v_ symmetry, whereas its ^1^A_1_ has a linear D_∞*h*_ symmetry. This crossing can be analyzed also through the spin and charge Mulliken population shown in Figures 4, 5. The triplet-singlet change has been obtained in the spin population (Figure 4) of OCO in the complex, which switches from the structure **BC**, in which the OCO has a bent geometry with high spin resembling the ^3^B_2_ state of CO_2_, into the **AC** complex in which the OCO is linear and has a low spin electronic distribution.

This crossing is associated with a sudden change in the electronic density: before the crossing the complex **BC** has a planar node in the wavefunction along the C-O_b_ bond, while after the crossing the planar node is transferred to the S-O_b_ bond in structure **AC** (see Figure 5). The second complex ***2*** has the minimum [OS–OCO]^+^ energy along the MEP with a value of −2.888 eV (−66.6 kcal mol^−1^), and a S-O_b_ distance of 2.3 Å. In this complex ***2*** the OCO has almost D_∞*h*_ symmetry, with the two C-O distances, C-O_b_ and C-O_c_, of 1.2 Å. After the crossing the high spin density is entirely located on the SO subsystem, with the S atom increasing its spin population up to that of the free SO^+^ +0.641 ℏ, whereas the spin population of the O_a_ reaches the value of +0.359 ℏ. Another interesting feature appearing both in Figures 4, 5 is the discontinuity in the spin and partial charge atomic population at the S-C distance of about 3.75 Å. Here there is a symmetry modification of the system, which changes from the C_s_ symmetry of the initial complex (see leftmost structure in Figure 3), to the C_2v_ symmetry of the [SO_2_–CO]^+^ complex ***1***. Indeed, at greater S-C distances the CO and SO_2_ are bound only via the S-O_b_-C atoms, whereas the interaction for shorter S-C distances is characterized by double O_a_-C and O_b_-C intermolecular forces. The C_2v_ symmetry of this part of the reaction is confirmed by the fact that the O_a_ and O_b_ atomic partial charges are almost equal in the region of the MEP that foregoes the first complex ***1***, and also in the region that follows the same complex almost up to the point of intersystem crossing at R_Ob−C_ of 1.55 Å.

**Figure 6:**
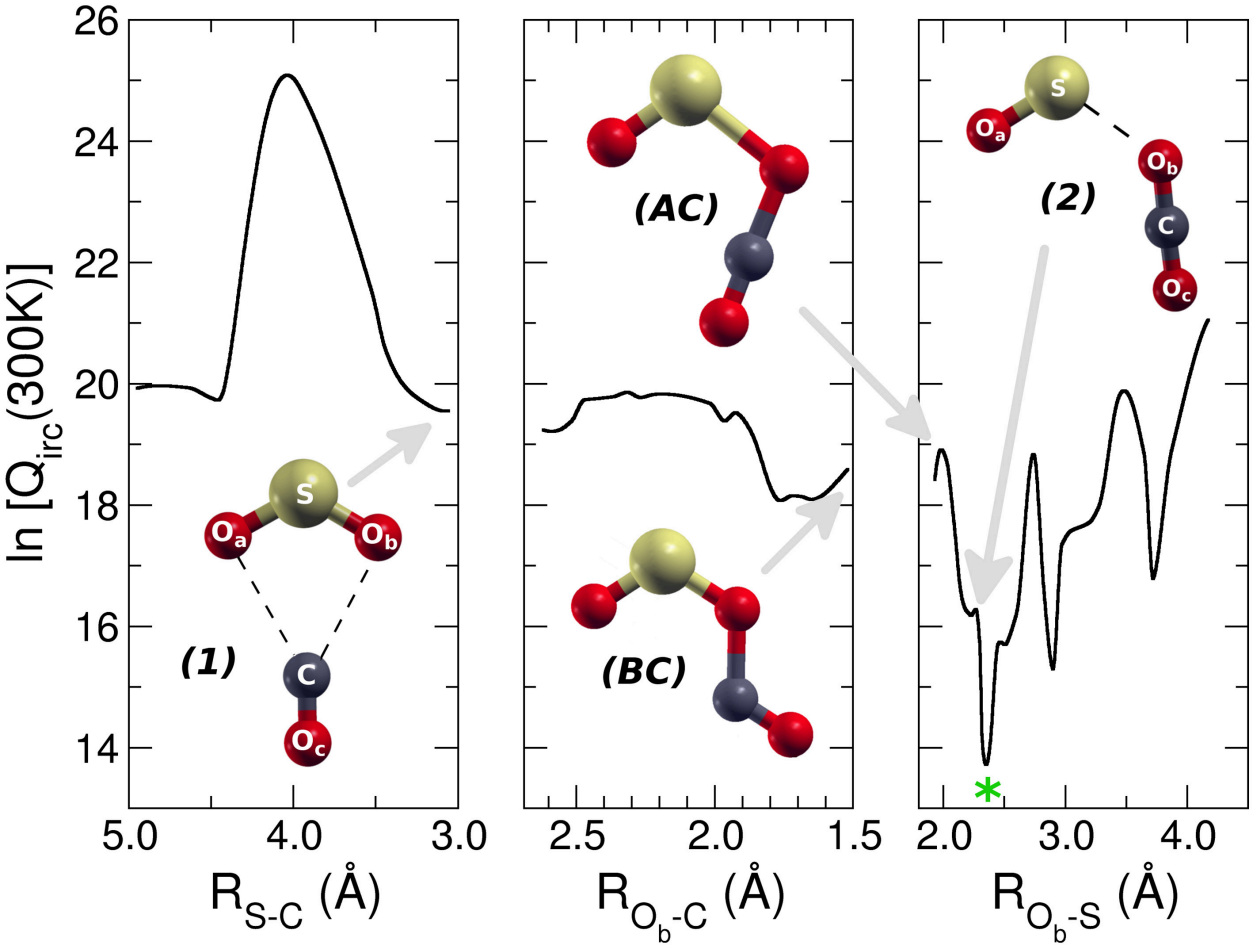
Molecular partition function of the adduct [SO_2_–CO]^+^ during the reaction at T = 300 K. The three panels correspond to the three reactive regions of the approaching reactants, intersystem crossing, and products formation. Complex (1), (2) and intersystem crossing are indicated with gray arrows. The green star indicates the location of the variational transition state (VTS).

Because of the barrierless nature of the MEP associated with the O transfer from ${\text{SO}}_{2}^{+}$ to CO, the VTST (Carelli et al., 2011; Satta et al., 2015b; Bao and Truhlar, 2017) is the computational model used to study the dynamics of this reaction. We have considered a molecular partition function Q_irc_ defined as the total molecular partition function with the vibrational part built over all the frequencies except the one relative to the internal reaction coordinate (IRC). The Q_irc_, including the zero point contribution is reported as a function of the three distances used to calculate the MEP of the reaction (Figure 6). However, the reaction coordinate ranges over which the Q_irc_ has been built is different from that of the MEP because otherwise it was not possible to define correctly the IRC frequency needed by VTST.

**Figure 7:**
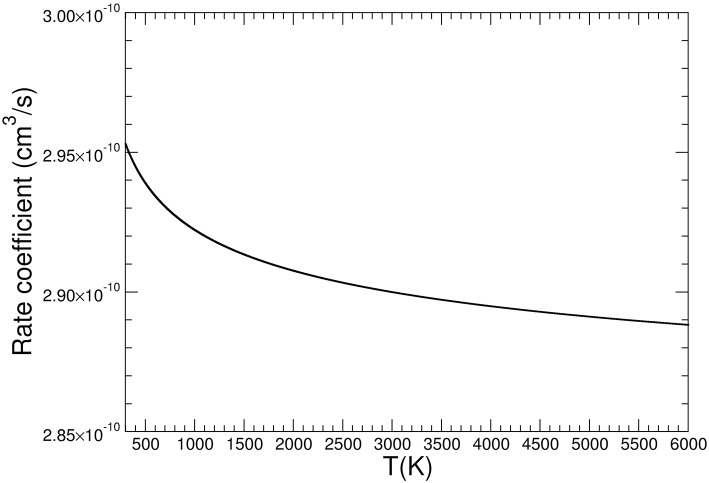
Calculated capture rate coefficient as a function of the temperature.

In the first approaching region (Figure 6 left panel), Q_irc_ increases and then rapidly decreases up to a minimum value corresponding to the formation of the first complex [SO_2_–CO]^+^ ***1***; in the second region (Figure 6 central panel) there is a first small barrier in the molecular partition function and then there is a decrease up to a double minimum at R_Ob−C_ of 1.7 Å. This is followed by a second barrier that brings very rapidly the system to the absolute minimum of the molecular partition function at R_Ob−S_ of 2.4 Å, corresponding to a geometry that resembles very strongly the [OS–OCO]^+^ complex ***2*** (Figure 6 right panel). The overall behavior of the molecular partition function at T = 300 K, with its absolute minimum in the region of the MEP that brings the system, after the intersystem crossing, from the more stable complex to the products, is such that the reaction has not a bottleneck up to the formation of the complex ***2***. Indeed this minimum corresponds to a dynamic state that resembles the products, and that cannot re-escape to the region of the reactants due to the energy barrier associated with the ^3^B_2_/^1^A_1_ crossing involving the C_2v_/D_∞*h*_ symmetry change of the forming CO_2_. The same qualitative behavior is observed for higher temperatures up to 6,000 K. The barrierless reaction has the VTST dynamic slowdown essentially in the region where the products are formed, hence the rate of the reaction can be determined by the capture theory. The simple Langevin formula for ion-molecule reaction predicts a constant rate coefficient with temperature, and since we aim to study the experimental decrease of the reaction probability with temperature, a capture collision rate constant based on trajectory calculations has been computed. In particular, we followed the parametrization of kinetic energy dependences of ion-polar molecule collision rate constants developed by Su (1994). This approach, which uses the dipole moment of the neutral reactant together with its polarizability, gives a temperature dependent rate coefficient *k*. In Figure 7 it is reported the *k* for a temperature range from 300 up to 6,000 K. Its value at 300 K is 2.95 × 10^−10^ cm^3^s^−1^molecule^−1^, in very good agreement with the experimental value of 3.00 × 10^−10^ ± 20% cm^3^s^−1^molecule^−1^ (Fehsenfeld and Ferguson, 1973). The increase of temperature determines a slow decrease in the rate coefficient, and at 6,000 K the rate of the reaction is only about 2% less than that at 300 K. This slow thermal trend is due to the quasi apolar nature of the CO molecule, which has a very low electric dipole moment (0.112 D). The model predicts that, at increasing temperature, the effect of the dipole moment of the neutral reactant on the effective interaction potential will be of weak decreasing strength: the thermal rotation average of the dipole moment will overcame the orientation effects induced by the charge of the ion. At variance, in the case of water, which has an electric dipole moment about 16 times greater than that of CO, the calculated rate coefficient decreases by a factor of 2.4 from 300 to 5,000 K (Cartoni et al., 2017). Even if we cannot directly compare the experimental SO^+^/${\text{SO}}_{2}^{+}$ ratio with the calculated rate coefficients, both the experimental and computational data show the same behavior: the reaction efficiency decreases with increasing energy content. The experimental trend is the same at the three pressures investigated, where almost the same decrease of about a factor of three is observed as a function of photon energy. A direct quantitative comparison between theory and experiments is not achievable because in the experiments the thermal condition could not be fulfilled.

The spin and charge trend calculated along the MEP, where effectively the spin and charge remain on SO^+^ moiety, thanks to the crossing point, could contribute to the high efficiency observed in this reaction.

## Conclusions

In this work a joint experimental and theoretical study of the ion-molecule reaction of the metal free sulfur dioxide ion ${\text{SO}}_{2}^{+}$ with carbon monoxide CO is reported. The “O” atom transfer reaction from ${\text{SO}}_{2}^{+}$ to CO is fast and efficient (ϕ = *k/k*_coll_ ≅ 1) and highly exothermic by about 45 kcal/mol, with an interesting reaction dynamics along the reaction path. Two energy minima are identified, [SO_2_–CO]^+^ and [OS–OCO]^+^ separated by an intersystem crossing barrier, with energy below that of the reagents, which couples the bent ^3^B_2_ state of CO_2_ with C_2v_ symmetry with ^1^A_1_ state with linear D_∞*h*_ symmetry. The spin and charge reorganization along the MEP are analyzed and eventually the charge and spin remain allocated to the SO^+^ moiety while CO_2_ molecule is rapidly formed. The values of the rate coefficient *k* at different temperatures are calculated with the capture theory. The value of 2.95 × 10^−10^ cm^3^s^−1^molecule^−1^is obtained at 300 K in very good agreement with the literature experimental value 3.00 × 10^−10^ cm^3^s^−1^molecule^−1^ ± 20%. The *k* values have a predicted negative trend with temperature, as also observed in the experiments. In the experimental conditions the formation of the weakly bound interacting collision complex is not favored at high internal energy of ${\text{SO}}_{2}^{+}$, probably due to an increase of the elastic scattering processes between non-thermalized reagents, namely a room temperature CO and a hyperthermal ${\text{SO}}_{2}^{+}$.

## Data Availability

The datasets generated for this study are available on request to the corresponding author.

## Author Contributions

AC planned the experiments. DC, AC, MC, PB, and ST contributed to the experiments. DC performed data analysis. MS performed the theoretical calculations. AC and MS wrote the manuscript. LA reviewed the manuscript. All authors gave their contribution to the discussion of the results and the preparation of the manuscripts.

### Conflict of Interest Statement

The authors declare that the research was conducted in the absence of any commercial or financial relationships that could be construed as a potential conflict of interest.
