# Supplementary material for: Gas Phase Oxidation of Carbon Monoxide by Sulfur Dioxide Radical Cation: Reaction Dynamics and Kinetic Trend With the Temperature
Source: Front Chem. 2019 Mar 26;7:140. doi: 10.3389/fchem.2019.00140 (PMC6443698; doi:10.3389/fchem.2019.00140)
Supplement: Supplementary file 1 [file Presentation_1.pdf]

Supplementary Material

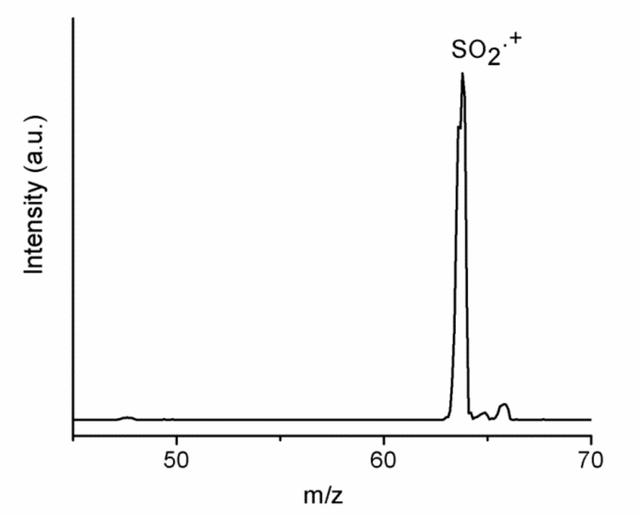

Figure 1S. Mass spectrum of SO_2 recorded at the photon energy of 14.0 eV, P_{SO2}=4.6x10^{-5}mbar, and without CO gas in the octupole (reaction zone).

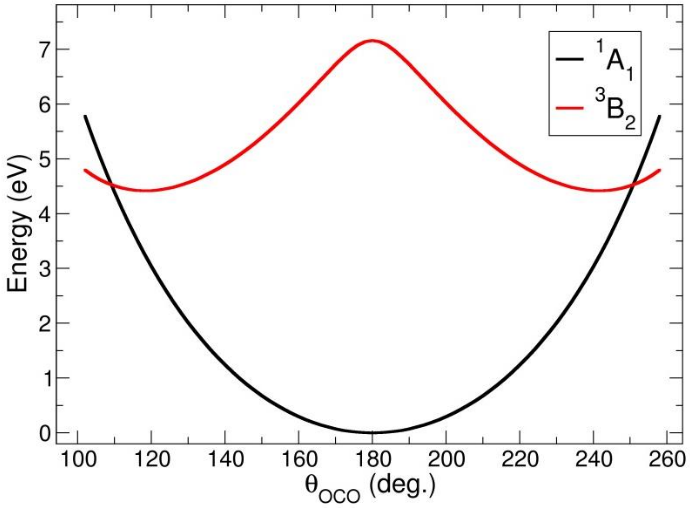

Figure 2S. OCO angle dependence of the triplet and singlet ground states for the isolated CO_2 molecule.

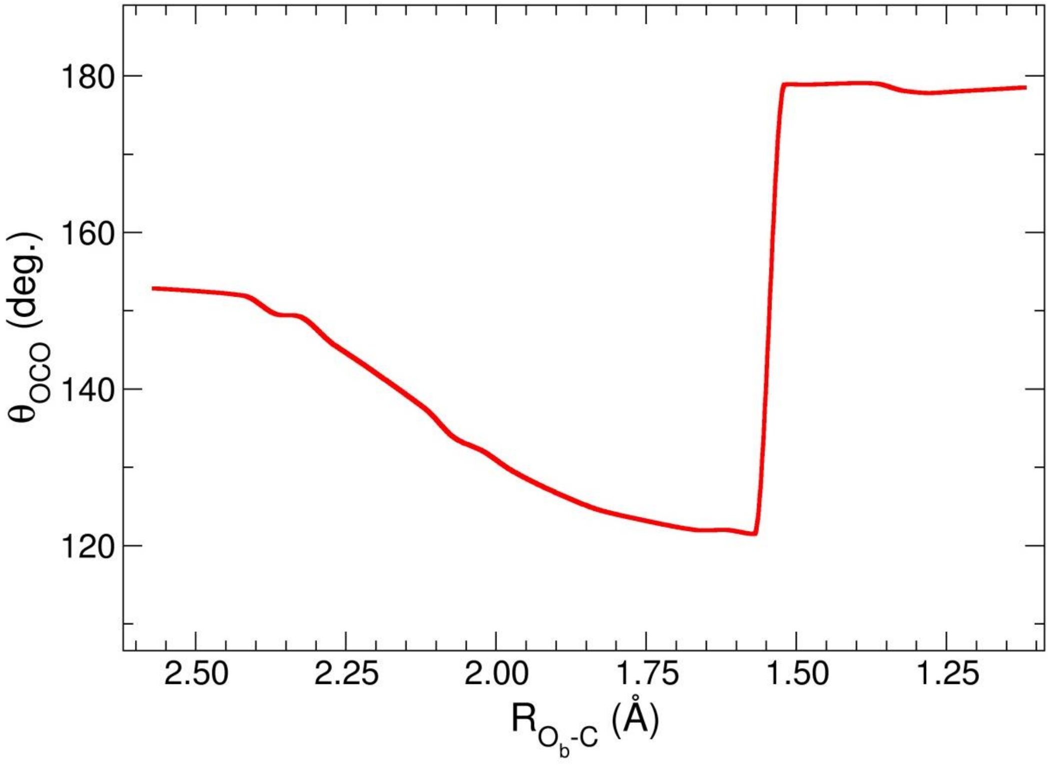

Figure 3S. OCO angle as a function of the reactive coordinate O_b-C along the MEP.

Discussion of Figures 2S and 3S:

The angle OCO is a good geometrical parameter to analyze and discuss the region around the intersystem crossing point. In particular when the reagents are approaching, left part of the central panel of Figure 3S, the angle OCO is about 150 deg. and it reaches a minimum of about 120 deg. just before the crossing occurs at RO_b-C=1.5 Å. After this point the angle OCO changes rapidly to a 180 deg. value, indicating that the CO_2 is almost formed in its linear configuration. A comparison of this trend with the angle OCO values of the singlet and triplet ground states of the isolated CO_2 molecule (Figure 2S), indicates that for values equal or less than 110 deg. the system is in its triplet state, whereas for angle greater than 110 deg. the CO_2 molecule is in its singlet ground state. Hence before the crossing point the CO_2 in the $[SO_2\text{--}OCO]^+$ adduct can be considered in its triplet state, but after that point the linear configuration of the OCO indicates that the singlet state is reached.
